# Translational challenges and opportunities in biofilm science: a BRIEF for the future

**DOI:** 10.1038/s41522-022-00327-7

**Published:** 2022-08-29

**Authors:** C. J. Highmore, G. Melaugh, R. J. Morris, J. Parker, S. O. L. Direito, M. Romero, F. Soukarieh, S. N. Robertson, N. C. Bamford

**Affiliations:** 1 NBIC Interdisciplinary Research Fellows, UK National Biofilms Innovation Centre (NBIC), Southampton, UK; 2 School of Biological Sciences, Faculty of Environmental and Life Sciences, University of Southampton, SO17 1BJ Southampton, UK; 3 School of Physics and Astronomy, University of Edinburgh, Edinburgh, EH9 3FD UK; 4 School of Engineering, University of Edinburgh, Edinburgh, EH9 3FD UK; 5 Biodiscovery Institute, School of Life Sciences, Faculty of Health and Medical Sciences, University of Nottingham, NG7 2RD Nottingham, UK; 6 Division of Molecular Microbiology, School of Life Sciences, University of Dundee, Dundee, DD1 5EH UK

**Keywords:** Biofilms, Antimicrobials, Applied microbiology

## Abstract

Biofilms are increasingly recognised as a critical global issue in a multitude of industries impacting health, food and water security, marine sector, and industrial processes resulting in estimated economic cost of $5 trillion USD annually. A major barrier to the translation of biofilm science is the gap between industrial practices and academic research across the biofilms field. Therefore, there is an urgent need for biofilm research to notice and react to industrially relevant issues to achieve transferable outputs. Regulatory frameworks necessarily bridge gaps between different players, but require a clear, science-driven non-biased underpinning to successfully translate research. Here we introduce a 2-dimensional framework, termed the Biofilm Research-Industrial Engagement Framework (BRIEF) for classifying existing biofilm technologies according to their level of scientific insight, including the understanding of the underlying biofilm system, and their industrial utility accounting for current industrial practices. We evidence the BRIEF with three case studies of biofilm science across healthcare, food & agriculture, and wastewater sectors highlighting the multifaceted issues around the effective translation of biofilm research. Based on these studies, we introduce some advisory guidelines to enhance the translational impact of future research.

## Introduction

**Fig. 1:**
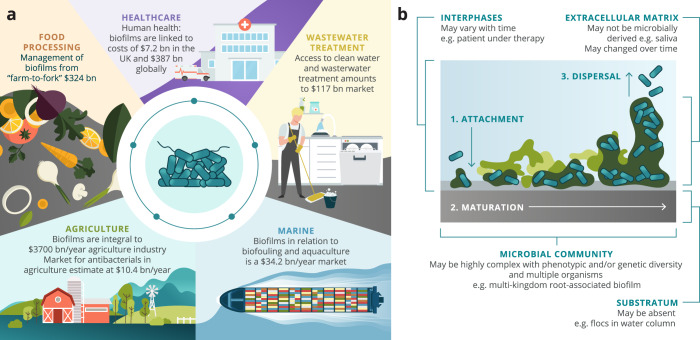
Biofilms impact on human activity and canonical model. **a** Outline of the scope and scale of biofilm interactions in human activity. Every facet of human health and the economy interacts with microbial biofilms. These span: food production (agriculture and aquaculture); food and fast-moving consumer goods processing; clinical applications to human and animal health; wastewater treatment and related environmental engineering; and all marine uses, including transport and resource extraction. The estimates of economic impact are from NBIC’s commissioned study and are available in their Annual Report 2021^72^ and recent publication^5^. **b** The canonically-understood colonisation-maturation-dispersal model of biofilms, and counterexamples. Biofilms are frequently modelled (in theory and in vitro) as single-species communities, which adhere to a physical substratum, colonising it, mature through extracellular matrix modelling and cell growth, to a climactic dispersal state. However, while this core model is well-understood and experimentally tractable, it frequently oversimplifies key aspects of real-life biofilms. These considerations are expanded on in the text around the image (black font). These include the substratum (which may be absent [as in water columns in WWT] or alter over time [as in a healing wound]); the community composition itself (which may contain multiple species, or even kingdoms, as in rhizobial communities including phage and fungi); the solution (which may vary rapidly, as in therapeutic antibiotic use) and the extracellular matrix (which may be partly or even wholly a result of non-microbial processes, as in saliva of the buccal cavity).

Biofilms are multicellular communities of microorganisms encapsulated in a matrix of exopolymeric substances^1^. They are diverse, ubiquitous, and are play a huge role in many diseases, and thus biofilms have been described extensively in the literature^2–4^. Biofilms can be both beneficial and detrimental to human society depending on their location and the species involved. One study on the international biofilm market found the estimated global economic impact of biofilms to be worth over $5 trillion USD annually^5^. The study covered the sectors of health, food security and safety, water security, antimicrobial resistance (AMR), climate change, and energy (Fig. 1a).

To study aspects of biofilms in the laboratory, researchers have developed various in vitro assays that attempt to mimic relevant environments, and theoretical models (computational or mathematical). These models are less complex than the biofilm system of interest and therefore allow researchers to make predictions that are reliable and repeatable. There is a multitude of experimental models, with many of the recent ones having been highlighted in a review by Guzman-Soto et al.^6^. Yet, laboratory results often fail to translate effectively, struggling to address ‘real-life’ situations in healthcare and industry. Furthermore, the canonical description of a biofilm is a simplification, there is a large diversity and greater complexity to biofilms found in societally relevant settings (Fig. 1b). For instance, dental biofilms vary with colonised substrata wherein implants, teeth, and mucosa can all play host^7^.

Due to knowledge-gaps in our understanding of how to prevent, detect, manage, and engineer biofilms for societal benefit, research consortia have formed around the world including: the National Biofilms Innovation Centre (NBIC) in the United Kingdom; the Center for Biofilm Engineering (CBE), USA; the Singapore National Biofilms Consortium (SNBC); Costerton Biofilm Centre (CBC), Denmark; among others. A major goal of these groups is to promote synergy between academia and industry to address unmet needs and grow economies. One step that has been taken is the formation of the ‘International Biofilm Standards Task Group’ that looks to develop international standardised biofilm test methods (info Box 1).

**Fig. 2:**
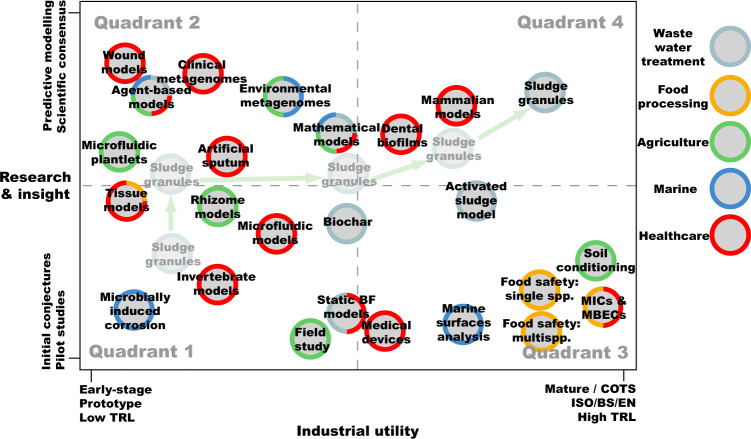
The BRIEF for evaluating and translating biofilm research. A selection of biofilm models, techniques, frameworks or applications derived from industry and academic basic science are presented on a two-dimensional plot. *Horizontal axis*: degree of technical maturity, scalability and/or deployability & standardisation of tools/approaches to monitor a biofilm system or application, from non-standardised, early-stage, prototype, low-Technology Readiness Level (TRL) approaches, to mature, commercially-available off-the-shelf (COTS), high-TRL approaches likely to have ISO, EN, DIN, BS, or other, standards within a defined regulatory framework. *Vertical axis:* depth and quality (predictive ability) of overall empirical understanding about the system. Colours correspond to applications relevant to wastewater treatment; food processing; food production (including agriculture and aquaculture); marine and petrochemicals; and human and animal healthcare. See Info Box 3 on Biochar. Supplementary Table 1 for more details^2,11,21,41–43,58,73–96^.

The gap between industrial practices and academic research is evident across the biofilms’ field. A key example is that many industrial standardised efficacy tests use planktonic microbes (e.g., CLSI Minimum inhibitory Concentration - Table 1). Unfortunately, planktonic tests have little relevance to the sessile microbes observed across many sectors. Translational research and the bridging between academia and industry has obvious benefits but there are multiple barriers. Identification and mitigation of these barrier can be difficult but will lead to mutually beneficial research and practices. To this end we have created a 2-dimensional framework, termed the Biofilm Research-Industrial Engagement Framework (BRIEF), for organising current biofilm technologies (including assays, practices, and models) relevant to biofilms across sectors, based on their accuracy to current science knowledge and industrial application (Fig. 2). The sectors we’ve focused on are healthcare, the food sector, agriculture, marine environments, and wastewater treatment.

**Table 1:** Examples of currently used standards for biofilm testing.

Number of standard	Description	Reference
**AATCC - American Association of Textile Chemists and Colorists**		
AATCC TM100 -2019	Test method for antibacterial finishes on textile materials	https://microchemlab.com/test/aatcc-100-antimicrobial-fabric-test/
**ASTM - American Society for Testing and Materials**		
ASTM E2196-17	Standard test method for quantification of *Pseudomonas aeruginosa* biofilm grown with medium shear and continuous flow using rotating disk reactor	https://www.astm.org/e2196-17.html
ASTM E2315-16	Standard guide for assessment of antimicrobial activity using a time-kill procedure	https://www.astm.org/e2315-16.html
ASTM E2562-17	Standard test method for quantification of *Pseudomonas aeruginosa* biofilm grown with high shear and continuous flow using CDC (Center for Disease Control) biofilm reactor	https://www.astm.org/e2562-17.html
ASTM E2647-20	Standard test method for quantification of *Pseudomonas aeruginosa* biofilm grown using drip flow biofilm reactor with low shear and continuous flow	https://www.astm.org/e2647-20.html
ASTM E2799-17	Standard test method for testing disinfectant efficacy against *Pseudomonas aeruginosa* biofilm using the MBEC Assay	https://www.astm.org/e2799-17.html
ASTM E2871-21	Standard test method for determining disinfectant efficacy against biofilm grown in the CDC biofilm reactor using the single tube method	https://www.astm.org/e2871-21.html
ASTM E3161-21	Standard practice for preparing a *Pseudomonas aeruginosa* or *Staphylococcus aureus* biofilm using the CDC biofilm reactor	https://www.astm.org/e3161-21.html
ASTM E3180-18	Standard test method for quantification of a *Bacillus subtilis* biofilm comprised of vegetative cells and spores grown using the colony biofilm model	https://www.astm.org/e3180-18.html
ASTM E3321-21	Standard test method for intraluminal catheter model used to evaluate antimicrobial urinary catheters for prevention of *Escherichia coli* biofilm growth	https://www.astm.org/e3321-21.html
ASTM E3151-18	Standard test method for determining antimicrobial activity and biofilm resistance properties of tube, yarn, or fiber specimens	https://www.astm.org/e3151-18.html
ASTM E645-18	Standard practice for evaluation of microbicides used in cooling water systems	https://www.astm.org/e0645-18.html
**ISO - International Organization for Standardization**		
ISO/DIS 4768	Measurement method of anti-biofilm activity on plastic and other non-porous surfaces	https://www.iso.org/standard/80309.html
ISO 16954:2015	Test methods for evaluating the efficiency of treatment methods intended to prevent biofilm formation or removal in dental unit procedural water delivery systems under laboratory conditions.	https://www.iso.org/standard/58009.html
ISO 11731:2017	Water quality - Enumeration of *Legionella*	https://www.iso.org/standard/61782.html

One dimension of the framework is the level of “scientific insight” (Fig. 2, y-axis), including understanding of the underlying model or system, ranging from early stages of research or correlative understanding (no mechanistic detail), to models with a common scientific consensus and thorough mechanistic detail. The second dimension is “industrial utility” (Fig. 2, x-axis) accounting for current industrial practices. Parameters such as scalability, standardisation, repeatability, and acceptance into common practices determine the position of the biofilm application on this axis. At the lower extreme of both axes (Quadrant 1), there is pilot or exploratory research, in which little is known and the potential for industrial application is not yet clear. The other extreme (Quadrant 4) contain societally beneficial technologies (or models) that are scientifically robust, well-understood, and effectively translated. Creating technologies and models that fit this category should be the goal of all translatable research. In many situations current practices do not satisfy these parameters.

Herein we cover three key case studies from three quadrants (Quadrants 2, 3 and 4) of our framework to better demonstrate the issues or barriers facing academics and industries. We propose that a Translationally Optimal Path (TOP) exists, when synergy and efficiency can be found through facilitated open communication, networking, cross-disciplinary collaboration, and open research. This must be sustained and reinforced over an extended period of time, supported by developing scientific consensus and regulatory alignment. We posit that long-term, multi-institution multidisciplinary research consortia, such as NBIC in the UK, will be key players in this effort: they can catalyse early-stage interactions between industry and academics; provide a mouthpiece for regulatory change needed to drive innovation; and can provide the supportive, long-term environment necessary to realise the industrial benefits of translated biofilm research (either by direct awards, seed funding, sandpits, or through convening power). Despite this, there are actions that can be taken by individuals to increase the translatability of their research. Below we explore three case studies and from these suggest guidelines to fellow researchers to aid in their quest for translatability and societal impact.

Box 1 Biofilm research consortia and International Standards Task Group Consortia have formed around the world centred on promoting biofilm research and knowledge. These consortia include the National Biofilms Innovation Centre (NBIC) in the United Kingdom (UK); the Center for Biofilm Engineering (CBE), USA; the Singapore National Biofilms Consortium (SNBC); and Costerton Biofilm Centre (CBC), Denmark. CBE, SNBC, and CBC all have research centres associated with their groups creating an environment for collaboration and knowledge transfer. CBE was the first centre to form with over 30 years of activity emphasising research, education, and industry collaboration. NBIC, formed in 2017, has instead taken a decentralised approach starting with four lead universities. One of their primary aims is to facilitate collaboration between academics and industry throughout the UK.

The CBE in Montana created a Standardized Biofilm Methods Laboratory with the mission of designing and testing systems and methods for the assaying the efficacy of biofilm control strategies. Their work has led to the acceptance of six biofilm methods by the American Society of Testing and Materials International (ASTM), these are listed in Table 1.

Leading biofilm research centres in the US, Europe and Asia are working together with a common goal to develop international standardised biofilm test methods. The group is called International Biofilm Standards Task Group and its mission is “to drive the international development and acceptance of standardized biofilm test methods in health care, the built environment and industrial systems.” The task group focuses on testing methods in health care settings, industrial systems, and the built environment to enable informed and consistent decision making on the international regulation of products targeting biofilm prevention and control. The group is open to input from industry and other agencies. Further information on the International Standards Task Group is available at both the NBIC and CBE websites^97^.

## Case study A – wound infection and treatment

Firstly, we consider areas of biofilm research that, in the BRIEF, are well understood with a common consensus and mechanistic detail but have had limited or poor translation into an industrial or clinical setting (Quandrant 2). For this case study, we will focus on wound biofilm models. Modern day wound treatment has an estimated annual cost of £8.3 billion to the NHS in the U.K^8^ and estimated Medicare cost of $96.8 billion in the U.S.A^9^. The NHS alone uses 250 million wound dressings a year with an average of eight different wound dressings used per patient^8^. While this figure includes general wound care, a large on-going cost is attributed to chronic wounds that are non-healing primarily due to biofilm related chronic infection. These long-term injuries carry significant morbidity, shortening active lifespan and curtailing economic participation for many. Their aetiology is complex, with biofilms associated with 78.2 % of chronic wounds according to one systematic review^10^. While some risk-factors for non-healing are appreciated, others are complex or poorly understood. Wound physiology itself is varied and complex; oxygenation gradient, nutrient availability and immune cells’ access all vary throughout the wound due to the interaction between biofilm structure, tissue type, body location, patients’ lifestyle and many other factors. Many in vitro biofilm-infected wound models have been developed to mimic chronic wound infections^11^. Collectively this would suggest that the significant body of scientific understanding pertaining to chronic wound modelling and treatment evaluation is ripe for industrial exploitation, yet this is not yet effectively translating to positive clinical outcomes.

### Context: current practice and standards

Translational research has mainly focused on wound treatment with a strong emphasis on antimicrobial wound dressings for the treatment of chronic wounds. Current testing standards to claim that a product possesses antimicrobial activity are performed using methodologies such as the AATCC 100–2019 antimicrobial fabrics test^12^ or the ASTM E2315 standard guide for assessment of antimicrobial activity using a time-kill procedure^13^. These methods only require activity against planktonic microbes, not biofilms, yet biofilm structures are observed in 78% of chronic wound^10^ and within 6% of acute wounds^14^. Currently, there is a disconnect between accepted standards and the role biofilms play in wound treatment and healing. Decades of research has focused on the development of wound biofilm models that recapitulate facets of the host environment to better represent the in vivo conditions, conditions that have been demonstrated to alter biofilm formation, structure, and the penetration of antimicrobials^15^.

### Development of biofilm-relevant models

Herein we describe some of the developments made in models biofilm infection of wounds for the purposes of framing translational barriers. A more technical review of relevant models was recently published by Thaarup et al.^11^. Wound biofilm models started with the adaption of simple standardised tests, a common example being the CDC biofilm device ASTM E2799^16,17^ and have progressed through to more novel in vitro models that mimic part of the host environment. The Lubbock wound model^18^ was one of the first to mimic the host by growing the biofilms in media containing plasma and red blood cells. In this model key wound microbes such as *Pseudomonas aeruginosa*, *Staphylococcus aureus* and *Enterococcus faecalis*, showed increased resistance to treatment as compared to standard tests of planktonic microbe cultures. Progression past the Lubbock model has included semi-solid models^19^, collagen-based models^20^ and modified collagen hydrogels. Townsend and colleagues^21^ developed a hydrogel based cellulose model containing *P. aeruginosa*, *S. aureus* and *Candida albicans*. This model demonstrated an increase in the biofilms’ tolerance to the topical antimicrobials chlorhexidine and povidone iodide, compared to bacteria treated in standard microtiter plates. The biofilm architecture differed in the 3D hydrogel model, where it was suggested that an increase in surface area led to higher biofilm cell counts in comparison to microtiter plate models. The biofilm constituent species were also less evenly distributed in the hydrogel model^21^. However, as the hydrogel model was predominantly based on non-mammalian derived compounds the structures observed may not be related to those observed in vivo^21^. And, many of these models use relatively ‘immature’ (12 h) biofilms to represent wounds that present as chronic in clinics (>4 weeks^22^) so further work to validate or refine these is clearly needed.

Tissue culture models have also been developed with the 2D wound scratch keratinocyte models (where keratinocytes constitute 90–95% of the upper endothelial layer) being used in combination with multiple microbial species to review how wounds heal when challenged with antimicrobial agents^23^. Two-dimensional models, however, fail to generate the biofilm structures found in vivo^23^. 3D Artificial dermal models have been considered and can be divided into two categories: Scaffold-free or Scaffold-based. Self-assembled skin substitutes (SASS) are scaffold-free and consist of a dermis layer generated with extracellular matrix produced by fibroblasts cultured with ascorbic acid, on which keratinocytes are cultured on top to form an upper epithelial layer. Scaffold-based models are comprised of human-derived fibroblasts within a dermal matrix. These 3D models can be used to investigate the wound milieu. The wound milieu plays an important but underappreciated role and is very hard to capture dynamically due to its varied and complex nature. Wound milieu models have been specifically developed to mirror i*n vivo* biofilm structure along with the increased antimicrobial tolerance associated with biofilm formation^24^.

Commercially available products that offer a full-thickness human skin equivalent, incorporating dermal and epidermal components have previously been used to investigate biofilm eradication by a novel compound^25^. This raises an interesting intersection between the development of new wound models with academic and industry buy-in (collaboration) versus the current commercially available and fee for service models.

### Translational challenges

These example models provide reliable in vitro options to test the efficacy of products and treatments, each with pros and cons (e.g., high-throughput vs biological ‘realism’). The models outlined above are generally low throughput and high cost compared to the planktonic assays currently approved for use. Thus, the translatability is still poor or limited, despite significant time and investment in this research, demonstrating that this field fits Quadrant 2 of our BRIEF (Fig. 2). Future collaboration between academia, wound-focused industry and regulatory bodies is needed to develop new in vitro models that better represent the wound environment and improve translatability. These collaborations would also serve to address the great variety of wound cases to ensure that wound biofilm models will effectively translate to specific clinical scenarios. Additionally, more suitable testing paradigms with appropriate model systems to challenge potential therapeutics are needed at various stages of investigation. Of particular importance is the development of in vitro models that can better model differential oxygenation status and the capability to run long term experiments >14 days – 1 month. This would better capitulate the biofilms seen in chronic infection^22^. Equally, the high cost, low-throughput nature of cutting-edge research models acts as an additional barrier to updake; academic scientists should strive develop models balancing realism with usability concerns for industry and regulators. Proprietary in-house models while useful, cannot by definition form a stable base for open regulatory standards and so are a potential obstacle to clinical translation of wound biofilm research; therefore, we advocate for collective working groups to be established and standardise these methodologies, via engaging with lobbyists. This will ensure reproducibility and lead to the development of approved standards for efficacy claims that will likely translate to better patient outcomes.

## Case study B – efficient fruit production

In Quadrant 3 of our framework lie methods, sensors, and techniques that are at a high Technology Readiness Level (TRL), or are Commercially available as Off-The-Shelf packages (COTS), and are routinely used by small and large industrial actors, clinical users and governmental stakeholders. They may even have nationally- or internationally-recognised standards and procedures associated with them (see Table 1). Interventions to manage and/or engineer biofilm applications and their conditions are likely to be well-developed and common across industries, and commercially available.

Despite this technical and procedural maturity, the fundamental science underpinning these applications is poorly understood or outdated. It may be that a problem has yet to be studied in detail using a mechanistic, empirical framework – one such example being the very limited understanding applied to biofilm communities in food processing plants, which are frequently treated through blanket cleaning and decontamination processes, despite the fact they are actually polymicrobial and complex^26^. Alternatively (as we shall see below), the scientific problem itself could be obvious in terms of motivation, but sufficiently complex that despite multiple lines of research (physiological, materials science, molecular), a broadly shared consensus has yet to form. As a result, the biofilm interaction at the system’s core is frequently conceptualised only as a ‘black box’.

Either way, there is little successful translation between what scientific understanding does exist of the system and impactful application to industry or the clinic. While understood commonly across an industry, interventions and best-practice fall within the customary practice (‘we operate this way, because other ways fail’) and thus, innovation through applied scientific understanding is challenging. Predicting the effect of altered or improved inputs or treatments may be impossible^27^.

### Context: current practice and standards

Let us illustrate this with an example. Microorganisms play an essential role in plant health, where biofilm formation is linked to microbial colonisation and persistence^28,29^. Intensive soft-fruit agriculture, such as greenhouse-enabled *Solanaceae* production (tomatoes, peppers and chillies) is a significant agricultural industry, with 16.4 M tonnes (approximate retail value: £80bn) produced intensively in EU in 2020^30^. Fruit production at this scale requires the engineered optimisation of multiple inputs in climate-controlled conditions, with parameters such as soil moisture and temperature, air humidity and temperature, pH, macro- and micro-nutrient levels all closely monitored in order to optimise yield and timed inflorescence (fruiting) while minimising labour. Bespoke subscription-based weather forecasting, soil quality surveys, and remote-sensing data, e.g., hyperspectral imaging, may also be used^31^. Additionally, costly resources are expended on managing these through heating, cooling, irrigating, fertilising, or treating crops^27^. However, there is no detailed understanding of the engine room driving plants’ nutrient uptake: the plant-biofilm interaction within the soil itself, widely recognised as essential in understanding and modelling plants’ growth^32^. The agriculture sector would benefit from understanding modulation and control of the microbial social communities to increase yield and lower costs during food production.

### Translational challenges

Despite the industrial need, an integrated scientific model predicting root-biofilm interactions and relating crops’ inputs to outputs is elusive, despite detailed understanding of root physiology^33^, the molecular mechanisms of nutrient uptake, numerous descriptions of the root-associated microbiome of common crops, both in the lab^34^ and in use^35^, as well as specific experiments on rhizobial biofilms themselves^36^. These have investigated, for example, plant-fungi^37^, bacteria-plant^28,29^, and bacteria-fungi interactions^38,39^ using advanced techniques such as transparent soil analogues^40^ and microfluidic modelling of microscale root-soil-biofilm interactions^41,42^. Complex multi-way interactions abound, such as suppression of the phytopathogen *Serratia plymuthica* by root-associated *B. subtilis* biofilms, which is enhanced by compounds released by the host plant^43^. In summary, the core problem itself is challenging, requiring multidisciplinary approaches to characterise, let alone manipulate or model, the genotypes, physiology and mechanisms of plants, microbes, soil, and pests^41,42^.

An improved understanding and predictive modelling ability could mitigate additional direct costs incurred by producers. For instance; phenology modelling with improved prediction of optimal harvesting dates might allow a grower to retain a labour force for half the number of days in a season, lowering costs^44^. Alternatively, improved understanding of the biofilm’s role in mediating nitrogen uptake might avoid over-fertilization (saturation)^45^, whereby too much nitrogen is added in the form of fertiliser in error, supressing yields and potentially encouraging pests and/or unproductive microbial passengers^46^. This last case also illustrates an additional positive societal externality, since excess N supplementation in crops is associated both with pollution of the aquatic and marine environments (through runoff and subsequent eutrophication) and promotes N_2_O release. N_2_O is a potent greenhouse gas, responsible for almost 300x the radiative forcing of CO_2_^47^, but again, precise modelling of N_2_O emissions is impossible, due to the issues mentioned.

What *concrete actions should we take* to ‘move industries up’ from Quadrant 3 to Quadrant 4 by generating, synthesising, and successfully applying knowledge? In fortunate circumstances (often where biological factors aren’t usually considered, such as food-processing plants), the problem, once articulated, may itself be scientifically tractable with low effort; these may be ‘low-hanging fruit’ where return on scientific investment is immediate. Here, improved collaboration networks and reciprocal awareness of industrial challenges and scientific capabilities have a good chance of succeeding.

But what about ‘hard’ scientific problems? Returning to the case study above, several features that complicate an already challenging task are apparent: Firstly, FAIR principles of data management are not followed often enough in practice^48^: this means while multiple datasets are collected, they are either proprietary and undiscoverable or else if released publicly and unified standards for metadata are gravely lacking (to take just ‘omics data resident in EMBL, 6,142 BioSample records for soil microbiome data exist, each with one or more accessions containing anywhere from 10Mbp to >1Tbp sequencing data. Labelling inconsistency between datasets means there are at least 658 discrete metadata keys defined in these metadata, illustrating the fragmented nature of these datasets)^49^. Efforts to establish community standards should accelerate, ideally with industrial support, and focus on translational (that is to say, industrial) goals. Secondly, data themselves may be collected ad-hoc, with the result that, for instance, soil temperature and air humidity timestamps are irregularly offset from each other and microbiome sampling. These would be easy to harmonise at a lower cost. Next, scientific communities (in public/basic and proprietary/applied science) may work in disciplinary silos, even with similar instrumentation, such that obviously complementary approaches are missed. For instance, two groups in the same city might be studying land use using hyperspectral imaging of remote sensing data; one might be estimating total climate impact through vegetation cover, another might be trying to optimise growth by detecting N or P deficiency. Crossing these disciplines may provide added value, while also promoting cross-fertilisation of ideas. Greater openness between research groups’ datasets could also accelerate innovative solutions to multidisciplinary biofilm problems. Finally, given the scale of the challenges, growers, buyers, public and private researchers and regulators should be open and transparent about the opportunities but also costs for failing to develop common, accurate, detailed models of rhizobial biofilm interactions that relate, e.g., fertiliser and climate inputs to crop yields, quality and inflorescence. In other words, the gains from sharing vast proprietary datasets may outweigh the costs in the long term.

## Case study C – wastewater treatment with Aerobic Granular Sludge

Finally, we consider biofilm technologies that have flourished through two-way engagement between academia and industry with the resulting synergy leading to highly translatable innovations that offer tangible societal benefits. This grouping is the top-right sector of Fig. 2 (Quadrant 4) and encompasses technologies with both high levels of scientific insight and industrial utility (e.g., oral biofilm models^50^ and wastewater treatment biofilms^51^). To illustrate how translatable biofilm research can move to Quadrant 4, we consider as an archetypal example the Nereda® Aerobic Granular Sludge technology.

### Context: current practice and unmet need

The conventional activated sludge (CAS) process is the most common type of wastewater treatment (WWT) used worldwide^52^. Each day, large volumes of influent wastewater are processed in municipal WWT works so that safe effluent is returned to the water cycle. Crucial to this process is the ability of bacteria to degrade dissolved pollutants in the wastewater whilst forming compact flocs that can be easily separated from the liquid. Although the CAS process is well established and has been the dominant type for WWT for over a century^52^, its long-term future^53^ is unfeasible due to poor settling of the CAS flocs, low biomass concentrations, and issues with effluent quality^54^. Furthermore, the CAS process requires separate tanks for aeration and settling, as well as separate processing units for carbon (C), nitrogen (N), and phosphorus (P) removal, which increases both the land and energy footprint of any treatment plant.

### Translatable solution

In the last few decades, the emergence of Aerobic Granular Sludge (AGS) technologies has provided a promising alternative to CAS^55^. Here, the microbes form large (>200 µm) smooth, spherical, granules (biofilms) with a much higher density than CAS flocs^56,57^. These granules possess excellent settling characteristics that give rise to higher quality effluent, as well as enabling aeration, nutrient removal (C, N, and P), and separation to take place within a single tank^55,58,59^.

### How AGS is applied

AGS technology employs sequencing batch reactors (SBR) to treat wastewater^60,61^. This is typically performed in three phases, with each phase imposing the necessary selection pressure to form dense and compact microbial granules composed of the appropriate spatial distribution of metabolic guilds^58^. Briefly, there is an anaerobic fill phase, which introduces nutrient-rich wastewater from the bottom of the tank. The wastewater passes through a settled bed of granules, whilst, owing to the plug-flow operations, pushes treated wastewater (from the previous cycle) out of the tank^62^. Then, there is the aeration phase^62^. Here, the granules are suspended in the tank due to the bubbling of air through the reactor, providing necessary dissolved oxygen to the granules. Finally, in the settling phase, the time allowed for sedimentation is sufficiently short that only granules with higher settling velocities are retained in the system^62^.

### How does it work?

The granules formed due to repeated cycles of these three phases, exhibit layered microenvironments that support a diverse microbial community and concomitant heterogeneous extracellular polymeric substances (EPS)^63–65^. The outer aerobic layer supports a community of nitrifying bacteria and contains EPS that is predominately comprised of polysaccharides, which help bind and stabilise the community. Due to microbial respiration and diffusion-limited oxygen availability, an anoxic/anaerobic zone exists in the interior. Here denitrifying, phosphate-, and glycogen-accumulating organisms are found^47,55,66^. At the very core of a granule is a highly anaerobic region and, in contrast to the EPS in the outer layer, the EPS in this core is protein rich. Thus, all the necessary biological conversions can be carried out in a single granule with excellent settling properties.

### Why it has been translated successfully

To better understand why the Nerada AGS technology has been a success story from both an academic and translational perspective, let us consider its evolution from fundamental lab-scale science. The study of microbial assembly into self-associated granules has been investigated since the 1960s and garnered increased interest in the 70 s and 80 s when they were incorporated as a component in anaerobic upflow sludge blanket reactors^67,68^. In the early 1990 s it was found that granules could be formed under aerobic conditions^69^ and researchers at TU Delft discovered a process by which granules could be generated in conditions common to most wastewater treatment operations^60,61^. Since then, the study of AGS has steadily grown and become an active area of research, as evidenced by the number of publications and review articles covering this expanding field of study^62^. That said, however, many aspects of granule formation are not yet fully understood (e.g., signalling, EPS mechanics, polymicrobial interactions), and so there is still huge scope for biofilm scientists to engage with this technology in the future.

The development of AGS was an outgrowth of a collaboration between academic researchers at TU Delft in the Netherlands and TU Munich in Germany. Research began in the early 90 s and culminated in a paper published in 1997 demonstrating a method for producing granular sludge under aerobic conditions^61^. The process was further refined through the late 90s, at which point a cooperative partnership was developed between researchers at Delft and Royal Haskoning DHV with further public support coming from the Dutch Water Board. Through this partnership, feasibility studies were initiated in 2000 and by 2003 a large pilot scheme was launched at the Ede Wastewater Treatment plant in the Netherlands. During this time, the academic researchers further refined and modified their process in order to translate from lab-scale to industrial-scale operations^55^. By 2006, municipal units were being constructed and 2010 saw the first full-scale municipal plant in operation. When compared to CAS, AGS technology requires 33% lower volumes^66^, up to 75% lower land footprint^58,62^, consumes up to 63% less energy^58,66^, and therefore reduces the overall operational cost by 50%^70^. It is not surprising, then, that within 25 years, Nereda AGS technology has become a well-established industrial process, with 89 full-scale installations spanning 18 different countries across 6 different continents^71^. Considering the variability in climate amongst these different locations, AGS technology is addressing global challenges for real wastewater treatment in both developed and developing nations. Furthermore, the largest Nereda plant in Ringsend Ireland serves 2,400,000 population equivalents and is one of the largest WWT plants in Europe^71^.

The translational success of AGS, from the lab to large scale global operations, can be attributed to several factors. First, wastewater treatment is a public health and environmental endeavour that is highly regulated. This means that new technologies face many hurdles to translate new ideas into full-scale deployment. In this case, the technology was relatively simple. The process could be achieved using established techniques and procedures within the wastewater industry meaning those hurdles become much reduced. Second, AGS allows for a nutrient removal process in one granule that achieves faster settling over standard activated sludge processes. This means fewer operating costs, more significant energy savings, improved effluent quality, and a smaller footprint for an AGS-based treatment plant than conventional sewage treatment plants. With the regulation of effluent standards becoming more stringent globally and ever-increasing energy costs, there is a solid economic and environmental case for AGS technologies. Third, there was a clear vision of how to translate this technology to a full-scale application from the very outset. The approach was to carefully consider how to scale the research even during the small-scale laboratory experimental phase. Fourth, this meant there was a clear roadmap to ‘sell’ the idea to the public and private sectors. A clear marketable plan helped facilitate the early coupling of academic research with public water authorities and private industry. And finally, a strong scientific understanding of the technology throughout its development enabled AGS to optimised with respect to parameter control and potential failures. These factors culminated in a translation that was fast by most standards: only 10 years from lab demonstration to the design and construction of the first municipal treatment facility.

## Conclusion

The large economic costs (Fig. 1a) associated with biofilm research suggests that there is huge potential for translational impact across many different sectors. Here we argue that such impact can result in tangible societal benefits when there is a high degree of two-way engagement between academia and the different sectors (e.g., AGS). Discussing the full impact of biofilms across sectors is beyond the scope of this review, instead, we attempt to categorise the many different biofilm-related technologies (and practices) used in both academia and industry (e.g., healthcare, agriculture, and wastewater treatment). In doing so, we present a framework in which these technologies are assigned positions in a two-dimensional space according to: 1 - the degree of scientific insight into the technology; and 2 – the degree of its industrial utility within or across various sectors. The resulting framework, BRIEF, provides a visual representation of the disparities found between fundamental research and industrial practices. Furthermore, as biofilm scientists, we strive to gain knowledge and a fundamental understanding of biofilm systems in many different environments. Doing so within the framework can help contextualise the translatability of our current research and identify future directions that maximise its positive impact and societal benefits.

Here we present our BRIEF in the context of three case studies highlighting the multifaceted issues around effective translation of biofilm research. These were selected from three different industries demonstrating the cross-sector importance and unique impacts biofilms have on society. Based on these studies we have some key recommendations for those wishing to increase the translational impact of their research. Case Studies A and B outline the multifaceted difficulties seen in biofilm translational research. Clinical and industry practices need to be standardised, scalable, reproducible, replicable, and cost effective. In Case Study A, biofilm wound models, we show that there are many advanced in vitro methods that have more accurate findings when exploring infection treatment, yet they lack the key traits required for industry use. Currently, there is no single accepted biofilm wound model and the standards in use today are at best rudimentary from a biological perspective. In some industries, the science unpinning the various biofilm technologies is not well understood. Case Study B provides a representative example of this situation. Biofilms play an integral role in crop yield, but owing to the large complexity of agricultural systems, a thorough understanding of this role seems beyond the current scope of agri-industry practices. In this instance, there is a pressing need for increased engagement and collaboration with academia in order to better understand agri-biofilms and to subsequently improve practices for optimising food yields and food security.

Case Study C is our example of efficient and effective biofilm technology translation. The Nereda AGS technology evolved from a pilot study to full-scale wastewater treatment solutions that have been implemented multinationally. By considering its history we see that the Nereda AGS technology involved a unified effort between academia, industry, and government, and we think that such high levels of cross-sectorial engagement at the conception of the technology facilitated its evolution along a Translationally Optimal Path (TOP) within our BRIEF (see Fig. 2). Therefore we make the following recommendations to guide research through a TOP and avoid the common pitfalls in academic engagement with industry:

### Examine the case for translation

Shifting from focused-academic research is not trivial, and imagining the benefits of possible applications is a necessary step to enact positive change. Collaboration offers opportunities to do exciting science, where successful translational technology requires established scientific pedigree. Industrial collaborations can act as catalysts for interdisciplinary research to face the cross-sector, multifaceted field of biofilm research.

### Early and open engagement

Engagement with industry can begin with pilot data at an early TRL, where a collaborative vision will better support translation. By reaching out to industry with speculative solutions to problems, the iterative process of refining the scientific approach with its realistic application can begin. This early stage is also the best time to navigate intellectual property (IP) issues (Info Box 2).

As the scientific understanding deepens, and potential industrial or clinical benefits become clearer, partnerships may become more formalised, perhaps exploring the opportunities together through joint funding applications. At this stage, all parties will want to be transparent and proactive. If necessary, new partners with slightly different needs might be sought; or else scientific questions revised to better address the industrial challenge.

Box 2 Intellectual property considerations Researchers may have concerns that early engagement with industry or third parties outside the academic sphere could lead to theft or inequitable exploitation of intellectual property (IP) such as patentable ideas, trade secrets, data, and knowhow. This concern is understandable but in practice unfounded. Multiple mechanisms exist to allow selective sharing of this property while protecting the originators, and most research institutions have funded and expert technology transfer officers able to guide academics through these. Non-disclosure agreements (NDAs) empower individuals and organisations to control what is, and isn’t, shared at a fine-grained level, or for a limited time or purpose; licenses can be used to share in potential current or future revenue; spin-out companies can act as vehicles for academic-industrial collaboration where funding, IP and expertise (research and business) can be pooled. When carefully managed, the opportunities that arise from early industrial engagement far exceed the risks to researchers.

### Incorporate good science into wider networks

Translational success in a mature academic-industrial partnership will rely on refinement and iteration of the application of science into the reality of the industrial setting. Through the iterative refinement process of translation, an abundance of an abundance of preliminary work not suitable for a particular application may be useful elsewhere. For greater and more equitable societal impact, data sharing is key. While we characterise industrial data as ‘closed’ and public science outputs as ‘open’, in practice many research datasets are functionally closed, i.e., hard to find, access, and integrate with other data. For true societal change, academic scientists should engage with lobbyists to facilitate regulatory frameworks that are responsive to scientific advances. If necessary, academia and industry can act jointly to build de facto standards.

### Utilise translation success

To realise translation of technology to the market or clinic, intensive academic collaboration is likely to be required over a period of years. Here, individual researchers’ contributions will shift from direct practical work to careful design, iteration, and validation. However, translation to industry is rarely the end of academics’ contributions, where large industrial-scale systems can offer exciting data opportunities for academics. Insights from successful translation can act as a foundation for future innovation, where the preliminary stages of the TOP have already been established.

Granted, not all practices and technologies must adhere to our TOP, which represent a guideline to translation for the individual researcher. We propose that it is possible for technologies and practices to move towards Quadrant 4 by other routes, where the principle of openness and communication are key to successful research translation. However, bridging connections between the right people, with the scientific background most suited to the industrial problems, is challenging owing to the shear breadth of biofilm research. This is where consortia like NBIC (UK), CBE (USA), SCELSE Centre (Singapore), and the EU Cooperation in Science Technology are needed. Through their growing networks and engagement practices, they will be instrumental at the research landscape level, establishing platforms to build collaborations, improving knowledge transfer, and facilitating in the optimal translation of biofilm-based technologies.

Box 3 Biochar and biofilms Biochar is a carbon-rich material produced by biomass pyrolysis in oxygen-limited conditions. Biochar uses range from reducing greenhouse gases emissions by sequestering atmospheric carbon, reducing soil nutrient leaching losses, increasing agricultural productivity, reducing the bioavailability of contaminants and soil remediation^98^. Recycled and reused Biochar-biofilm consortia showed viable economic long-term application in anaerobic digestion for waste or wastewater treatment^99^. The use of Biochar for soil improvement may have originated >8,000 years ago in the Brazilian Amazon^100^ rainforest, but only recently has been actively researched and exploited. Biochar is an excellent example of product standardization^101^ and active engagement between academia and industry.

## Supplementary information

Supplementary Table 1

## Data Availability

This is a review article with no produced or synthesised data.
