# Supplementary material for: Translational challenges and opportunities in biofilm science: a BRIEF for the future
Source: NPJ Biofilms Microbiomes. 2022 Aug 29;8:68. doi: 10.1038/s41522-022-00327-7 (PMC9424220; doi:10.1038/s41522-022-00327-7)
Supplement: Supplementary file 1 — Supplementary Table 1 [file 41522_2022_327_MOESM1_ESM.pdf]

| Case study | Decription of technology/model | Quadrant | Sectors | Industrial applicability | | Knowledge & insight | | Key ref(s) |
|---|---|---|---|---|---|---|---|---|
| | | | | Rank | Justification | Rank | Justification | |
| **Tissue models** | Co-cultured eukaryotic cell lines with different microbial species to allow a clearer understanding of host/biofilm interactions, simulate the human host environment more accurately than high throughput setups, and fill gaps identified in current animal models that do not reflect human physiology and disease. These advanced in vitro models recreate various biofilm-infected sites such as respiratory epithelia, mucosal tissue, chronic wounds via dermis biofilm models, and so forth. | Q1 - low-readiness and pilot studies | Healthcare, food processing | 2 - Low-med | These models allow to more closely mimic complex tissue structures and investigate therapeutic interventions. These setup lack the high throughput capabilities of simpler in vitro models | 3 - Medium | Careful co-culture choice and setup design along with key parameters, such as microbial and cell seeding densities and selection of analysis methods have to be considered. | (1, 2) |
| **Microbially-influenced corrosion (MIC)** | Modelling corrosion of material that is accelerated or influenced by resident microbes. | Q1 - low-readiness and pilot studies | Marine | 1 - Low | The application of treatments, preventions, and analysis of MIC in terms of the biofilms themselves is not widespread. Most methods are not informed by the microbes involveed. Reproducability and undersstanding are still lacking. | 1 - Low | Research is being done in this field but understanding of the realtionship between microbes and outcomes is lacking. Language inconsistances are also an issue with microbiologiest and electrochemists usin divergent terminology. | (3) |
| **Rhizome models** | Creation of model mixed bacterial community to mimic argicultural microbiomes. | Q1 - low-readiness and pilot studies | Food production | 2 - Low-med | Successful application is uncertain due to unknowns about variation between environments, cost of implimentation, and the question of whether to customise for every crop and geographical zone, and farmer adoption. | 3 - Medium | Analysis of microorganisms present in soil and in the rhizosphere is common. Samplings and genotyping is now routine. Understanding of the functions of each community member is also growing. | (4, 5) |
| **Invertebrate models** | Using insect models such as Galleria mellonella, Caenorhabditis elegans and Drosophila melanogaste rapid infection models can be performed in addition to not coming under use of animals in science. | Q1 - low-readiness and pilot studies | Healthcare | 2 - Low-med | Investigation of candidate theraputics, adjunctive therapies and analysis of toxicty can be performed in these models. These can be performed in a rapid low cost screening but require technical expertise. | 2 - Low-med | These models provide excellent early insight but lack standardisation and in cases hetrogenatiy in the communities can lead to non-significant results e.g. wax worms. Virulence factors of microbes can be species specific which can complicate adjunctive therapies | (6) |
| **Microfluidic models** | Microfluidic technologies permit the engineering, at the microscale, of controlled and varied environments and are particularly suited to studying how hydrodynamic forces can influence biofilm growth in such environments. | Q1 - low-readiness and pilot studies | Healthcare | 2 - Low-med | Often may not be the appropriate scale for industrial or clinical applications. Moreover, another issue with microfluidic systems is that they are effectively two-dimensional and an ongoing challenge will be to create more realistic microfluidic models that capture the network architecture and 3D chemical environments seen in the real world. | 3 - Medium | Microfluidic approaches have been very effective in elucidating mechanisms of biofilm growth and dynamics in complex environments, particularly under flow conditions. | (7) |
| **Field study** | Semi-controlled treatment of agricultural field as compared to control field. | Q1 - low-readiness and pilot studies | Food production | 3 - Medium | Setup is industry friendly as little change needs to be made to agricultural method. Farm output can be sacrificed in order to test new products and thus there are incurred risks. | 1 - Low | The inability to control many variables makes it hard to get more than just correlative findings. Anaysis of the biofilm communities is at endpoints and not dynamic. | (8) |
| **Static biofilm models** | Referring to agar plate colony assays and pellicle assays in test tubes (non-microtitre plate). | Q1 - low-readiness and pilot studies | Healthcare, WWT, Food production | 3 - Medium | These assays can be done cheaply. The throughput is lower than multi-well plate assays with length of analysis increased. | 1 - Low | These assays can elucidate qualitative biofilm traits and interactions with compounds/treatments but often don't correlate well with the real world due to oversimplification. | (9) |
| **Biochar** | Biochar-biofilm consortia as fertiliser and water treatment method | Q1 - low-readiness and pilot studies | WWT | 3 - Medium | Optimisation for markets and determination of product life cycle is needed. Investment by industries into creation of a robust reliable product is still needed. | 3 - Medium | Knowledge of how microbes interact with biochar has grown with multiple papers exploring their interaction. More work must be done to fully understand the interplay between biochar and resident microbes. | (10) |
| **Wound models** | These models intend to mimic certain aspects of the wound environment from nutrients avilable, microbes present and the wound milieu | Q2 - mature science but low readiness | Healthcare | 1 - Low | These have been extensively characterised in the academic setting and have been used to investigate theraputics and wound dressings in industry. These models can be complex and in cases reproducability can be an issue | 5 - High | See case study 1 | (11) |
| **Clinical metagenomes** | Predictive models of microbial communities found in clinical biofilms such as for lung, gastrointestinal and urinary mucosa, as well as at wound, implant and medical device sites. | Q2 - mature science but low readiness | Healthcare | 1 - Low | Although clinical metagenomic analyses and data are now common in research, few have resulted in successful translation to predictive medical applications such as (a) targetted, personalised antimicrobial treatment, or (b) introduction of commensal species to manage microbial communities probiotically. | 4 - High-med | Existing studies are frequently hard to find and integrate to metananalyses. Predictive models are necessarily limited by data availability, or anchored on specific clinical conditions of dysbiosis in study treatments that provide poor control/baseline data. | (12) |
| **Artificial sputum** | CALLUM | Q2 - mature science but low readiness | Healthcare | 2 - Low-med | The utility of artificial sputum models is limited in industry, where a lack of consensus on composition and variability, call into question the need for artificial sputum models in comparison to other basic, but low fidelity models such as MICs. | 3 - Medium | Presence of non-surface adhered aggregates is acknowledged but rarely incorporated into models. The composition of AS varies between research groups, which leads to different expression profiles of pathogens (P. aeruginosa). | (13) |
| **Mathematical models** | Different mathematical and computational models (e.g. continuum, discrete) have been developed with varying complexity to capture the growth and dynamics of biofilms in a variety of natural and industrially relevant contexts. | Q2 - mature science but low readiness | Healthcare, Food processing, Marine, WWT | 3 - Medium | The first efforts to model biofilm growth used simple 1-D continuum models to help understand biofilm development in industrial contexts such as wastewater. There have been great advances in developing more realistic and accurate models of biofilm development, but there still exists a gap between theoretical modelling and practical applications in industry. | 4 - High-med | Modelling approaches require significant technical knowledge and expertise that can span physics, biology, mathematics, computer science, chemistry, and engineering. As such, translating modern approaches and knowledge to practical and industrial settings can be a challenge. The communication of the efficacy and advantages of this approach can be challenging to non-experts and as such, represents a barrier to real-world application. | (14, 15) |
| **Microfluidic plantlets** | Using custom microfluidic chips with sprouting seeds to look at bacterial biofilms at the root surface | Q2 - mature science but low readiness | Food production | 1 - Low | The setup is custom and is low throughput. | 3 - Medium | These experiments allow for investigation of biofilm dynamics over a multiday timescale which has been a challenge in bacterial-plant interactions. There are only two publications using the technique so far so there is room for growth in the field. | (16, 17) |
| **Environmental metagenomes** | Predictive models of microbial communities found in environmental and industrial samples such as WWT, energy pipelines, agriculture, aquatic and marine ecosystems. Such predictive models would allow targetted and efficient deployment of antimicrobials while minimising development of resistance or tolerance; and/or indirect management of environmental and industrial biofilms through addition or promotion of key beneficial species or strains. | Q2 - mature science but low readiness | Food production, Marine | 2 - Low-med | There is an expanding number of commercially-available kits to collect and prepare soil samples for metagenomic analyses, but few open SOPs exist and price per sample is not commercially viable except for very large specialist breeders. Standards do not exist. | 4 - High-med | A large and growing body of public and private metagenomes collected represent substantial datasets (exabyte scale), although they have not been synthesised into coherent models with the power to predict crops' response to environmental change due to root-associated biofilm. | (18) |
| **Medical devices** | Use of in vitro biofilm models for the study of biofilm formation on antimicrobial surfaces. | Q3 - commercial-scale application but shallow science-base | Healthcare | 3 - Medium | Biofilm formation in medical devices is a huge worlwide concern, that could lead to severe cases of blood stream infections, sepsis or even death. Industry has an active interest in the development of new antimicrobial surfaces and effective cleaning protocols (e.g. cleaning of endoscopes). | 1 - Low | There is still very little knowledge what triggers microorganisms to form biofilms, especially in complex environments and clinically relevant settings. Organisms used often in models have altered surface attachment properties, culture media used is rarely representative. Many developed antimiicobial surfaces also fail to translate to clinical settings due to safety issues and the lack of in vitro biofilm models capable of long term study of antimicrobial activity. | (19) |
| **Marine surfaces analysis** | Analysis of the efficacy of engineering surfaces and treatments to control biofilm growth in marine environments. | Q3 - commercial-scale application but shallow science-base | Marine | 4 - High-med | Microfouling due to biofilm-forming microbial organisms can condition surfaces to enable attachment and colonisation of large organisms (e.g. barnacles, mussels) that impact sailing ships' hydrodynamic efficiency. Furthermore, microbial biofilms can induce corrosion in metal pipes and other metallic structures, posing a significant issue to the oil & gas industry, among others. Many engineering approaches can ameliorate these issues (e.g. surface coatings, paints, biocides, electrolytic systems). Despite these solutions, the cleaning and replacement of industrial components (e.g. ship hulls, pipes, membranes) still represent a significant cost to the operator. | 2 - Low-med | Modern '-omics' based approaches have revealed the extremely complex consortia of organisms that make up a marine biofilm. Moreover, the community composition can vary widely geographically and be dependent on local conditions, seasons, and weather. The inherent complexity of these issues poses a significant challenge to understanding and modelling these complex communities from a biological perspective. There have been numerous strategies that have been developed to prevent biofouling and biocorrosion within the laboratory setting such as the design of proteases, quorum sensing inhibitors, and the use of bacteriophages. A key issue is not necessarily the efficacy of these approaches but the scalability, both technical and economical, of these new technologies. | (20) |
| **Activated sludge model** | These models capture the kinetics and stoichiometry, in the form of mass balance equations, of the most important biological processes in the activated sludge process (e.g., bacterial growth and nutrient removal). Although ASMs do not consider spatial heterogeneity in bacterial biomass and nutrient concentration, they are nevertheless very useful for process engineers owing to their facile integration in WWT simulators (e.g. AQUASIM and BioWin). | Q3 - commercial-scale application but shallow science-base | WWT | 4 - High-med | Activated sludge models have been indispensable for the WWT plant design and optimisation, as well as providing WWT operators with mechanistic insight when problems arise. They are implemented in a lot of commercially available software, but need updating in order to better understand emerging problems, N2O emission. There is also a lack of integration with more complexbiological data, e.g., omics. | 3 - Medium | ASMs are now rather mature and although they have been extended to describe P, N, and COD removal, they are difficult to validate experimentally. Thus ASM are rather limited with respect to further developments. Furthermore, although the strength of AS model lies in their simplicity, they are now less fashionable and garner less interest compared to more sophisticated fields of study, e.g., omics. | (21, 22) |
| **Food safety: single-species** | Use of single species models | Q3 - commercial-scale application but shallow science-base | Food processing | 4 - High-med | The food industry uses various materials which can be colonised by biofilms and due to their frequent use, these make highly appropriate model surfaces for studying biofilms. Studies of biofilm formation of mostly single species have been done in different types of food industry relevant surfaces (e.g. stainless steel, polystyrene, glass...). | 2 - Low-med | Biofilm research in food environments has mostly focused on understanding the mechanisms and factors controlling biofilm formation in single or dual-species biofilm models. While only very limited information is available on the real architecture and composition of natural biofilms colonizing equipment and food processing environments. However, tools for biofilm sampling and in situ analysis in food industries have been developed. | (23, 24) |
| **Food safety: multispecies** | Use of multiple species models (usually 2 or 3 species) | Q3 - commercial-scale application but shallow science-base | Food processing | 4 - High-med | In the food industry, surfaces and equipment are often contaminated by microorganisms forming biofilms. The food industry has developed besides well defined cleaning and disinfection protocols, new promising early-stage detection and real-time monitoring techniques for biofilms. | 1 - Low | In vitro biofilm studies on Interactions of foodborne pathogenic bacteria with other food-related bacteria in dual- and multi-species biofilm models, have only been extensively studied in recent years. However, most in vitro models do not account the 3D structure of the polymicrobial biofilms, which has been shown to be quite relevant knowledge especially for biofilm eradication | (24, 25) |
| **Minimum inhibitory concentration assay** | Determination of the minimum amount of antimicrobial required to achieve no visible growth of a select microbe using planktonic growth conditions. | Q3 - commercial-scale application but shallow science-base | Food processing, Healthcare | 5 - High | Biofilm eradication is often easier to achieve in a microtiter plate than in a more advanced model. MICs principally deal with planktonic cultures despite the ubiquity of biofilms in the modelled environment. | 2 - Low-med | MICs focus principally on the effect of an antimicrobial substance on a microbial inoculum. The biofilm phenotype is not taken into account and rarely incorporated into the model. Whie MICs provide a highly standardised methodology to test antimicrobial compounds, they sacrifice the nuance and complexity of the in situ clinical or industrial environment, making them poor indicators of true efficacy. | (26) |
| **Soil conditioning** | Precision, real-time manipulation of soil conditions (abiotic factors such as temperature, chemistry and gas content, and biota e.g. root-associated biofim community composition) enabled by detailed predictive models of community composition. | Q3 - commercial-scale application but shallow science-base | Food production | 5 - High | Key geochemical measurements of soil condition (pH, temperature, humidity etc) are regularly undertaken using standardised and highly cost-effective kits by growers across the world, and/or subcontracted to specialist consultants. This information is actioned into treatments to manipulate and/or stabilise soil condition. | 2 - Low-med | The scientific understanding of how soil condition, rhizosphere, and plant root-microbiome (biofilm interface layer) interact to promote growth or resist disease is weak, and limited to largely model species in tightly controlled conditions. | |
| **Dental biofilms** | Simple to complex polymcrobial models and integration with human cell line to investigate host-pathogen response | Q4 - commercial-scale application of mature science | Healthcare | 4 - High-med | These models have extensively been used to investigate the effectivness of mouth washes and new potential addatives. In addition the models have also been used to investigate new dental materials and addatives applied to them | 4 - High-med | Stable biofilms with several species represented (>10). A well characterised and understood microbiome. The host-pathogen interaction and immunological effects can also be investigated easily. These polymicrobial communities are also stable. | (27) |
| **Mammalian models** | The most closely related models to humans ranging from mice through to rabbits. These models are regulated to varying degrees globally. | Q4 - commercial-scale application of mature science | Healthcare | 4 - High-med | These models provide critical information prior to clinical trials, they are well regulated and controlled studies. They are expensive and require high technical expertise. These models tend to be mono-species. | 4 - High-med | For theraputic development they provide key toxicology data but with a caveat that this may be different across species and moving towards humans. They provide key insight on theraputic bioavailability. | |
| **Sludge granules general** | *Dense compact granules, containting all necessary microbes for effective treatment of wastewater, with excellent settling and separation characteristics.* | Q4 - commercial-scale application of mature science | WWT | 5 | *There are ~90 full scale plants world wide.* | 4 | Although there is a high degree of academic engagment, there is still more scope to gain more fundamental insight and mechanistic understanding in such systems. | (28, 29) |
| **Agent-based models** | *Agent-based models (AGMs) have been used extensively to simulate bacterial biofilm formation on surfaces. In short, bacteria possess their own time-dependent state variables (e.g., position, growth rate, and size), which change subject to local nutrient conentration fields, which themselves change as a result of consumption and diffusion. Cell growth and division follow, and the macroscopic properties of the biofilm emerge from the microscopic interactions between cells.* | Q2 - mature science but low readiness | No specific sector, although there has been some focus on metabolic guilds found in WWT | 1 | Although ABMS provide funamdental insight into aspects of biofilm formation in certain industrial contexts, e.g., spatial stucture in infections and in wastewater treatment, interest is mainly academic. | 4 | There is a high degree of academic engagment in Biofilm ABMS from a range of disciplines, e.g., Physics, Computational Science, Biology, Mathematics, Environmental Engineering. Although, the models are often rather generic, simulations reveal how macrocopic behaviours and structure emerge from the microscopic interactions between the cells. | (30, 31) |

| **Quadrants** |
|---|
| Q1 - low-readiness and pilot studies |
| Q3 - commercial-scale application but shallow science-base |
| Q4 - commercial-scale application of mature science |
| Q2 - mature science but low readiness |

| Research and insight | |
|---|---|
| 1 - Low | Pilot studies and early exploratory work |
| 2 - Low-med | |
| 3 - Medium | Developing consensus and/or only partially predictive understanding of system |
| 4 - High-med | |
| 5 - High | Mature field; scientific consensus on detailed predictive understanding of system |

| **Industrial application and scalability scale** | |
|---|---|
| 1 - Low | Low technology-readiness; models only work prototype form or research lab context |
| 3 - Medium | Proof-of-concept or technology demonstrator prototypes but not commercially widely adopted |
| 5 - High | Mature technology, standards and or operating concepts; COTS solutions available and viable on the market |

References


1 Mountcastle, S. E. *et al.* A review of co-culture models to study the oral microenvironment and disease. *J Oral Microbiol* **12**, 1773122, doi:10.1080/20002297.2020.1773122 (2020).
2 Vyas, H. K. N., Xia, B. B. & Mai-Prochnow, A. Clinically relevant in vitro biofilm models: A need to mimic and recapitulate the host environment. *Biofilm* **4**, doi:ARTN 100069 10.1016/j.bioflm.2022.100069 (2022).
3 Little, B. J. et al. Microbially influenced corrosion—Any progress? Corrosion Science 170, doi:https://doi.org/10.1016/j.corsci.2020.108641. (2020).
4 Bell, T. H. *et al.* Factoring Ecological, Societal, and Economic Considerations into Inoculant Development. *Trends Biotechnol* **37**, 572-573, doi:10.1016/j.tibtech.2019.02.009 (2019).
5 Kaminsky, L. M., Trexler, R. V., Malik, R. J., Hockett, K. L. & Bell, T. H. The Inherent Conflicts in Developing Soil Microbial Inoculants. *Trends Biotechnol* **37**, 140-151, doi:10.1016/j.tibtech.2018.11.011 (2019).
6 Cutuli, M. A. *et al.* Galleria mellonella as a consolidated in vivo model hosts: New developments in antibacterial strategies and novel drug testing. *Virulence* **10**, 527-541, doi:10.1080/21505594.2019.1621649 (2019).
7 Yawata, Y., Nguyen, J., Stocker, R. & Rusconi, R. Microfluidic Studies of Biofilm Formation in Dynamic Environments. *J Bacteriol* **198**, 2589-2595, doi:10.1128/JB.00118-16 (2016).
8 Chowdhury, S. P. *et al.* Effects of Bacillus amyloliquefaciens FZB42 on lettuce growth and health under pathogen pressure and its impact on the rhizosphere bacterial community. *PLoS One* **8**, e68818, doi:10.1371/journal.pone.0068818 (2013).
9 Ogran, A. *et al.* The plant host induces antibiotic production to select the most-beneficial colonizers. *Applied and Environmental Microbiology* **85**, e00512-00519, doi:doi:10.1128/AEM.00512-19 (2019).
10 Jayakumar, A. *et al.* New directions and challenges in engineering biologically-enhanced biochar for biological water treatment. *Sci Total Environ* **796**, doi:ARTN 148977 10.1016/j.scitotenv.2021.148977 (2021).
11 Thaarup, I. C. & Bjarnsholt, T. Current In Vitro Biofilm-Infected Chronic Wound Models for Developing New Treatment Possibilities. *Adv Wound Care (New Rochelle)* **10**, 91-102, doi:10.1089/wound.2020.1176 (2021).
12 Chiu, C. Y. & Miller, S. A. Clinical metagenomics. *Nat Rev Genet* **20**, 341-355, doi:10.1038/s41576-019-0113-7 (2019).
13 Neve, R. L., Carrillo, B. D. & Phelan, V. V. Impact of Artificial Sputum Medium Formulation on Pseudomonas aeruginosa Secondary Metabolite Production. *J Bacteriol* **203**, e0025021, doi:10.1128/JB.00250-21 (2021).
14 Wang, Q. & Zhang, T. Y. Review of mathematical models for biofilms. *Solid State Commun* **150**, 1009-1022, doi:10.1016/j.ssc.2010.01.021 (2010).
15 Dzianach, P. A., Dykes, G. A., Strachan, N. J. C., Forbes, K. J. & Perez-Reche, F. J. Challenges of biofilm control and utilization: lessons from mathematical modelling. *J R Soc Interface* **16**, doi:ARTN 20190042 10.1098/rsif.2019.0042 (2019).
16 Noirot-Gros, M.-F. *et al.* Functional Imaging of Microbial Interactions With Tree Roots Using a Microfluidics Setup. *Frontiers in Plant Science* **11**, doi:10.3389/fpls.2020.00408 (2020).
17 Massalha, H., Korenblum, E., Malitsky, S., Shapiro, O. H. & Aharoni, A. Live imaging of root–bacteria interactions in a microfluidics setup. *Proceedings of the National Academy of Sciences* **114**, 4549-4554, doi:doi:10.1073/pnas.1618584114 (2017).
18 Buhmann, M. T., Stiefel, P., Maniura-Weber, K. & Ren, Q. In Vitro Biofilm Models for Device-Related Infections. Trends in Biotechnology 34, 945-948, doi:https://doi.org/10.1016/j.tibtech.2016.05.016 (2016).
19 Gomez-Alvarez, V. in *Microbial Biofilms: Methods and Protocols* (ed Gianfranco Donelli) 323-340 (Springer New York, 2014).
20 de Carvalho, C. C. C. R. Marine Biofilms: A Successful Microbial Strategy With Economic Implications. *Frontiers in Marine Science* **5**, doi:10.3389/fmars.2018.00126 (2018).
21 Gujer, W. Activated sludge modelling: past, present and future. *Water Sci Technol* **53**, 111-119, doi:10.2166/wst.2006.082 (2006).
22 Sin, G. & Al, R. Activated sludge models at the crossroad of artificial intelligence-A perspective on advancing process modeling. *Npj Clean Water* **4**, doi:ARTN 16 10.1038/s41545-021-00106-5 (2021).
23 Fernández-Delgado, M. *et al.* BIOFILM FORMATION OF Vibrio cholerae ON STAINLESS STEEL USED IN FOOD PROCESSING. *Rev Inst Med Trop Sao Paulo* **58**, 47, doi:10.1590/s1678-9946201658047 (2016).
24 Alvarez-Ordonez, A., Coughlan, L. M., Briandet, R. & Cotter, P. D. Biofilms in Food Processing Environments: Challenges and Opportunities. *Annu Rev Food Sci T* **10**, 173-195, doi:10.1146/annurev-food-032818-121805 (2019).
25 Strathmann, M., Mittenzwey, K. H., Sinn, G., Papadakis, W. & Flemming, H. C. Simultaneous monitoring of biofilm growth, microbial activity, and inorganic deposits on surfaces with an in situ, online, real-time, non-destructive, optical sensor. *Biofouling* **29**, 573-583, doi:10.1080/08927014.2013.791287 (2013).
26 Townsend, E. M. *et al.* Development and characterisation of a novel three-dimensional inter-kingdom wound biofilm model. *Biofouling* **32**, 1259-1270, doi:10.1080/08927014.2016.1252337 (2016).
27 Welch, J. L. M., Rossetti, B. J., Rieken, C. W., Dewhirst, F. E. & Borisy, G. G. Biogeography of a human oral microbiome at the micron scale. *P Natl Acad Sci USA* **113**, E791-E800, doi:10.1073/pnas.1522149113 (2016).
28 Zheng, S., Lu, H. & Zhang, G. The recent development of the aerobic granular sludge for industrial wastewater treatment: a mini review. *Environmental Technology Reviews* **9**, 55-66, doi:10.1080/21622515.2020.1732479 (2020).
29 Nancharaiah, Y. V. & Sarvajith, M. Aerobic granular sludge process: a fast growing biological treatment for sustainable wastewater treatment. Current Opinion in Environmental Science & Health 12, 57-65, doi:https://doi.org/10.1016/j.coesh.2019.09.011 (2019).
30 Jayathilake, P. G. *et al.* A mechanistic Individual-based Model of microbial communities. *PLoS One* **12**, e0181965, doi:10.1371/journal.pone.0181965 (2017).
31 Lardon, L. A. *et al.* iDynoMiCS: next-generation individual-based modelling of biofilms. *Environ Microbiol* **13**, 2416-2434, doi:10.1111/j.1462-2920.2011.02414.x (2011).
